# Driving Behavior of Older and Younger Drivers in Simplified Emergency Scenarios

**DOI:** 10.3390/s25165178

**Published:** 2025-08-20

**Authors:** Yun Xiao, Mingming Dai, Shouqiang Xue

**Affiliations:** 1 School of Urban Construction and Transportation, Hefei University, Hefei 230606, China; dmm2286088075@163.com (M.D.); xuesq@hfuu.edu.cn (S.X.); 2 Anhui Provincial Key Laboratory of Urban Rail Transit Safety and Emergency Management, Hefei 230601, China

**Keywords:** older drivers, driving performance, eye movement, driving characteristic, emergency traffic situations

## Abstract

This study focuses on exploring the differences in driving abilities in emergency traffic situations between older drivers (aged 60–70) and young drivers (aged 20–35) in a simple traffic environment. Two typical emergency scenarios were designed in the experiment: Scenario A (intrusion of electric bicycles) and Scenario B (pedestrians crossing the road). The experiment employed a driving simulation system to synchronously collect data on eye movement characteristics, driving behavior, and physiological metrics from 30 drivers. Two-factor covariance analysis, correlation analysis, and regression analysis were conducted on the experimental data. The comprehensive study results indicated that the older group exhibited better driving performance in emergency scenarios compared to the younger group. Specifically, in Scenario A, the older group had a faster first fixation time on the AOI compared to the younger group, a faster braking reaction time, a higher maximum brake pedal depth, and a higher skin conductance level. In Scenario B, the older group’s driving performance was similar to that in Scenario A, with better performance than the younger group. The study reveals that in some simple driving tasks, young-old drivers (60–70 years) can compensate for their physiological decline through self-regulation and self-restraint, thereby exhibiting safer driving behaviors.

## 1. Introduction

In 2023, the population aged 60 and above in China reached 296.97 million, accounting for 21.1% of the total population, indicating that China has entered a moderately aging society [1]. As of the first half of 2022, the number of drivers aged 60 and above in China numbered 18.82 million, with an average annual increase of approximately 1.5 million from 2017 to 2022 [2]. With the growing scale of older drivers, their driving safety issues have gradually become a focal point in the transportation field. Many scholars have conducted research on the driving characteristics of older drivers.

Numerous studies have demonstrated that driving performance is influenced by behavioral control, visual perception, psychological factors, and cognitive load [3]. Carmeli et al. [4] pointed out that with increasing age, the total amount of skeletal muscle, strength, and mobility of older people decline, which adversely affects driving control. Zhang et al. [5] compared the decision-making and response times of older and middle-aged/younger drivers using light signals as stimuli and found that with increasing age, drivers’ perception time, judgment time, and action time are prolonged. Guo et al. [6] investigated the takeover behavior of drivers in autonomous driving scenarios and discovered that older drivers have slower reaction times to takeover requests compared to younger drivers. Liu et al. [7] compared the stress responses of older and middle-aged/younger drivers under different risk conditions and found that older drivers have longer reaction times in taking measures. Bieliauskas et al. [8] found that in complex driving environments with multiple tasks, such as turning, judging traffic conditions, and parking, the coordinated control ability of older drivers is affected, leading to extended reaction times. Yoshitake et al. [9] studied the reaction time and pedal error rate of older and younger drivers when performing six acceleration or braking operations with one or both feet and found that older drivers are more prone to pedal errors as the task difficulty increases.

The above studies demonstrate that physiological degradation may cause older drivers to be slow and even make mistakes. During the driving process, vision and hearing are both crucial for safe driving. In fact, most of the relevant information is acquired through visual behavior while driving [10]. Owsley et al. [11] indicated that with increasing age, drivers’ visual sensitivity decreases, and their ability to pay attention in multiple directions is impaired. Key et al. [12] compared the visual perception abilities of younger and older drivers while watching driving video clips and found that older drivers have weaker visual perception abilities than younger drivers. Biernacki [13] investigated changes in visual activity in older drivers over 65 years of age with increased cognitive load and found that as the task difficulty increased, the older drivers’ scanning and fixation time became longer, and the efficiency of visual transfer decreased rapidly. Maud Ranchet [14] explored the impact of age on the detection of vulnerable road users (VRUs) and found that VRU detection performance declines with age. Scott et al. [15] compared the gaze of novice drivers, younger skilled drivers, and older drivers passing right-turn intersections and found that the older drivers’ gaze was more concentrated and their sweep was smaller. Guo et al. [16] independently developed an effective visual field test method and tested the effective visual field of younger, middle-aged, and older drivers (38 in total). Through differential analysis, they found that drivers’ visual field capabilities deteriorate significantly with increasing age.

Additionally, statistical data and research by scholars indicate that older drivers are more likely to be involved in traffic accidents or casualties. According to the National Highway Traffic Safety Administration, in 2022, the traffic fatality rate per 100,000 population for individuals aged 65 and above was 13.79, compared to 12.47 for those under 65 [17]. Statistics from Japan show that from 2005 to 2014, the proportion of traffic accidents involving older drivers nearly doubled (from 10.9% to 20.4%).

Although most studies suggest age-related degradation in driving abilities among older drivers, scholars have increasingly recognized their compensatory strengths: Lisa et al. [18] indicated that older drivers exhibit greater psychological stability, heightened safety awareness, and enhanced emotional regulation. Qiu [19] demonstrated that drivers in early-stage cognitive decline maintain preserved driving safety competencies. Chen et al. [20] confirmed that older drivers initiate earlier deceleration responses during lane-changing scenarios, exhibiting superior cognitive driving capacities and cautionary behaviors compared to younger cohorts.

We note that most studies suggesting a significant decline in the driving ability of older drivers were conducted under complex driving conditions, and few compare the driving characteristics of young-old drivers (60–70 years) and younger drivers in simple traffic conditions.

Based on these findings, we hypothesize that young-old drivers (60–70 years) may mitigate the adverse effects of physiological decline in simplified driving environments. To validate this proposition, we designed a driving simulation study on a 20 km straight road. Given that China’s substantial e-bike population is a principal contributor to traffic accidents and that pedestrians crossing roads is a common cause of traffic accidents [21,22,23], two sequentially presented emergency scenarios were designed: e-bikes and pedestrians entering the vehicular lane. Utilizing a driving simulator, an eye-tracking device, and physiological instruments, driving simulation experiments were conducted to simultaneously collect driving behavior data, eye movement data, and physiological data from both young-old drivers (60–70 years) and younger drivers (20–35 years). The study employed two-factor analysis of covariance (ANCOVA), correlation analysis, and regression analysis to examine driving characteristics of two groups of drivers during emergency situations in simplified traffic conditions.

## 2. Materials and Methods

### 2.1. Participants

**Figure 1:**
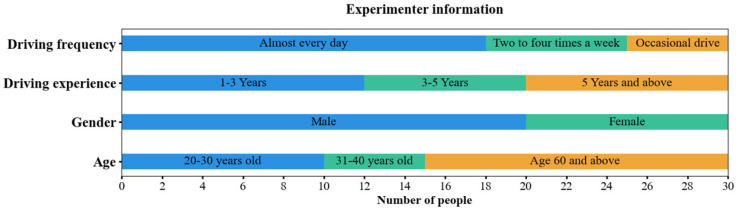
Participants’ information.

The study recruited a total of 30 licensed healthy drivers and divided them into two experimental groups: the older group (15 individuals) and the younger group (15 individuals). The gender distribution was balanced, with 10 males and 5 females in each group. In the older group, there were 6 people with over 5 years of actual driving experience, 6 with 3–5 years, and 3 with 1–3 years; in the younger group, 4 drivers had over 5 years of actual driving experience, 2 had 3–5 years, and 9 had 1–3 years. The younger group ranged in age from 20 to 35 years, with a mean age of 27 years. The older group ranged in age from 60 to 70 years, with a mean age of 65 years. All participants had at least one year of driving experience and possessed proficient driving skills; moreover, all participants had normal visual function that met the requirements for safe driving. Detailed information regarding the participants is presented in Figure 1. Participants were asked to get plenty of sleep in the 24 h before the experiment and to not consume alcohol, caffeine, or any functional drinks that may affect the nervous system.

### 2.2. Experimental Equipment

The primary experimental equipment used in this study includes a driving simulator, the ErgoLAB Human–Machine–Environment (HME) Synchronized Cloud Platform, SCANeR Studio 2021.1 simulation software, and physiological monitoring devices. The specific details are as follows:

ErgoLAB HME Synchronized Cloud Platform: This is an integrated system engineering tool that combines scientific, integrated, and intelligent features. It allows for the synchronized acquisition and comprehensive Human–Machine–Environment analysis of data from human, machine, and environment interactions. In this experiment, the platform was used to synchronously collect electrodermal activity (EDA) signals, eye movement characteristics, and driving behavior data to analyze the participants’ driving behaviors.

**Figure 2:**
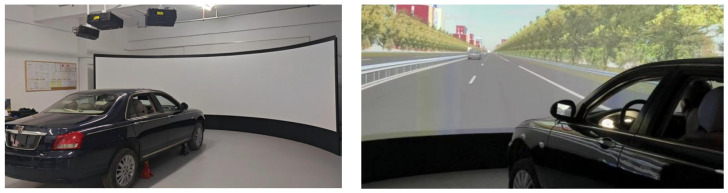
The driving simulator platform and the process of the experiment.

Driving simulation platform (Kingfar Technology Inc., Beijing, China): The driving simulation platform consists of a modified automatic transmission car simulator (sampling rate of 100 Hz), SCANeR Studio 2021.1 simulation software (AVSimulation, Paris, France), and a projection system. SCANeR Studio 2021.1 is a specialized software for automotive and traffic simulation, providing all the necessary tools and models to build highly realistic virtual worlds. It can create a highly realistic 3D simulation environment and offers strong modularity and expandability. The visual scene was projected onto a dedicated curved screen system. The projection screen was a hard, curved screen measuring at least 2.25 m (height) × 5 m (width) with a radius of curvature ≥ 2.5 m. The screen offered a horizontal viewing angle of 160 degrees, with uniformity >95% and surface roughness ≤ 25 μm. The screen is capable of displaying complex scenes rendered at up to 1 million polygons and 30 fps to ensure visual fluidity. The experimental procedure using the driving simulator platform is shown in Figure 2.

Tobii Pro Glasses 2 (Tobii AB, Stockholm, Sweden): These are a high-precision eye-tracking device used to record changes in drivers’ eye movements during the driving process. They capture real-time gaze trajectories and pupil fixation information, with a sampling frequency of 50 Hz.

ErgoLAB Physiological Sensor (Kingfar Technology Inc., Beijing, China): This device employs wearable technology combined with 2.4 GHz wireless radiofrequency transmission technology to capture physiological data from participants in real-world conditions (sampling rate of 64 Hz), ensuring minimal interference and constraints.

### 2.3. Experimental Scenarios

**Figure 3:**
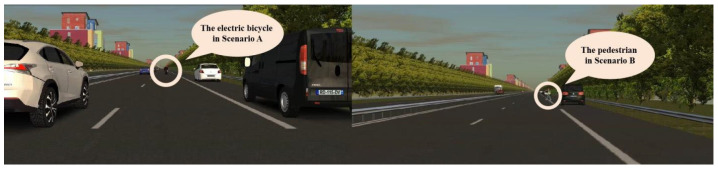
Scenario A and Scenario B.

The study employed a two-factor repeated-measures design with age group as a between-subjects factor. Given the widespread use of electric bicycles as a primary mode of short- to medium-distance travel in China, the study designed two types of emergency scenarios to investigate the differences in driving characteristics between older and younger drivers in mixed-traffic scenarios: e-bike intrusion and pedestrian crossing. The experimental scenario included the following components: road section design → roadside landscape design → script design → traffic flow design. The specific settings are as follows:

The scenarios were set during dusk hours (18:00–19:00), and the experimental road section was a straight, two-way, six-lane highway with a design speed of 60 km/h. The road was equipped with a central reservation, green belts, and a moderate number of building models. The road alignment was a straight line, without any special alignments such as long downhill slopes, sharp bends, or steep inclines. To minimize external interference, the curtains in the driving laboratory were kept closed during the experiment, and the brightness of the projection screen was adjusted to an appropriate level. The designs of Scenario A and Scenario B are shown in Figure 3.

Scenario A: E-bike intrusion. During the normal driving process of the experimental vehicle, the system triggered an electric bicycle intrusion event. Specifically, an electric bicycle suddenly entered the motor vehicle lane of the experimental vehicle from the far-right lane, obstructed from the driver’s view, simulating the common behavior of e-bikes illegally occupying lanes. Scenario A was set at the one-third point of the experimental road section, located 6.6 km from the starting point, lasting about 3 s.

Scenario B: Pedestrian crossing. During the normal driving process of the experimental vehicle, the system triggered a pedestrian crossing event. Specifically, a pedestrian moved from the right side of the road to the left side at a constant speed of 4.5 km/h (1.25 m/s). The pedestrian’s trajectory was perpendicular (90°) to the direction of the experimental vehicle’s travel, simulating a typical scenario of a pedestrian illegally crossing the road. Scenario B was set at the two-thirds point of the experimental road section, located 13.2 km from the starting point, lasting about 3 s.

### 2.4. Experimental Procedures

The participants’ personal information, including their name, gender, age, and actual driving experience, was collected. Each participant was provided with an introduction to the experiment, instructions on the use of the driving simulator, and details regarding the experimental procedure and safety precautions. These instructions encompassed an introduction to the mechanical components of the driving simulator, compliance with traffic rules, maintaining the middle lane, adhering to the speed limit (60 ± 5 km/h), spontaneously reacting to changes in the driving environment, and the privacy protection of participant data. Then, participants entered the vehicle to familiarize themselves with the driving simulator. They drove on a stable adaptation segment of the road for 10 min to become accustomed to the simulator. Once comfortable, they proceeded to sequentially put on the experimental equipment. The eye tracker was put on, and its connection and calibration were performed; once calibrated, the system began recording eye movements. Participants also wore an electrodermal activity (EDA) sensor. To ensure high-quality EDA signals, electrodes were securely affixed to the participant’s left index and middle fingers, while the signal receiver was stabilized on the left upper arm via an elastic strap. Once the experimental equipment was properly worn, the participants got into the vehicle to start the experiment. They drove continuously on the designated experimental road until passing the marked endpoint, during which the eye tracker and EDA sensor continuously collected data. After the participants exited the vehicle, we first turned off the experimental equipment. Second, we checked and confirmed the validity of all the data. Third, we ruled out simulator sickness or stress in the participants. Following these steps, the experiment was concluded. After the experiment, all the data collected during the experiment was analyzed using ErgoLAB 3.0.

### 2.5. Ethics Statement

All methods were carried out in accordance with relevant guidelines and regulations. All experimental protocols including questionnaires were approved by experts. The experiment was conducted in the Driving Simulation Laboratory of Hefei University, and all participants knowingly and voluntarily participated in the experiment. We confirm that informed consent was obtained from all participants. The results of the study will be used only for academic research, and it did not have any negative impact on the participants.

### 2.6. Research Metrics

#### 2.6.1. First Fixation Time on Area of Interest (AOI)

The first fixation time on the AOI is when the participants first direct their gaze to a specific area of interest during the experiment. During the period from the onset to the offset of each emergency scenario, we positioned the AOI as a rectangular region centered on the hazard source to measure the attention mechanism of participants.

**Figure 4:**
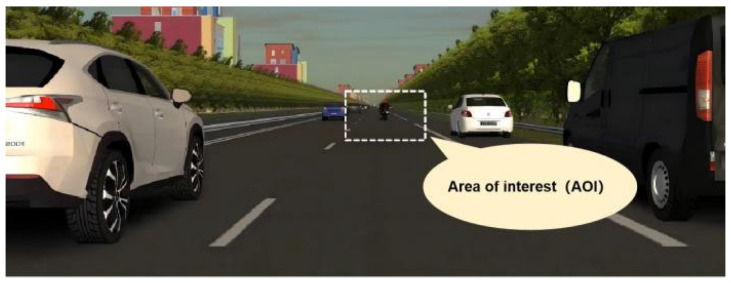
Area of interest (AOI).

Eye movement events were classified using the I-VT (Identification by Velocity Threshold) algorithm in ErgoLAB 3.0. This method calculates the angular velocity between consecutive gaze points, classifying points with a velocity ≤ 30°/s as potential fixations. Sequences of such points lasting ≥ 100 ms were merged into fixation events. Finally, fixations within predefined AOIs were extracted to compute the first fixation time and proportion of fixation duration. The meaning of the first fixation time on the AOI is presented in Table 1, and the setting of the AOI is shown in Figure 4.

#### 2.6.2. Proportion of Fixation Duration on AOI

This is the percentage of the total fixation time that participants spend looking at a specific area of interest. This measure indicates the relative attention allocated to particular regions, reflecting their engagement or importance during the task. The meaning of the proportion of fixation duration on the AOI is presented in Table 1.

#### 2.6.3. Driving Behavior Metrics

During the obstacle avoidance process, the manipulation of the brake pedal is divided into four stages: the perception and recognition phase, the judgment and manipulation phase, the braking manipulation adjustment phase, and the braking manipulation withdrawal phase [24]. This study compares the total duration of the perception and recognition phase and the judgment and manipulation phase between two groups of drivers, referred to as the braking reaction time. Additionally, the maximum depth of the brake pedal refers to the deepest extent to which the brake pedal is pressed by the driver during the driving process. This metric quantifies the degree of pedal displacement, reflecting the driver’s force and habits during braking. In emergency scenarios, a greater maximum depth of the brake pedal indicates that the driver tends to decelerate more quickly and thoroughly, demonstrating a more cautious and conservative driving style. The meanings of braking reaction time and maximum brake pedal depth are presented in Table 2.

#### 2.6.4. Physiological Data Metrics

Electrodermal activity (EDA) is an electrical phenomenon that occurs with the activation of sweat glands. When the sympathetic nervous system is activated, sweat gland activity is enhanced, leading to an increase in skin surface conductivity. EDA is one of the most sensitive metrics for measuring emotional states and can directly reflect the activity level of the sympathetic nervous system, which is associated with states such as alertness, emotional arousal, and cognitive load [25]. The meaning of mean EDA is presented in Table 3.

## 3. Results

All data collected was synchronized and preprocessed using ErgoLAB software, thereby obtaining all test data from both groups of drivers. Through correlation analysis, potential associations among driving behavior, eye movement, and physiological indicators were examined. With driving experience as a covariate, the differences in driving performance between the two groups of drivers were explored through analysis of covariance (ANCOVA) and regression analysis.

### 3.1. Correlation Analysis

The results revealed significant correlations among these variables. First, the brake pedal reaction time and first fixation on the AOI demonstrated a significant positive correlation (Scenario A: r = 0.741, *p* < 0.01; Scenario B: r = 0.739, *p* < 0.01). Second, there was a significant negative correlation between the brake pedal reaction time and brake pedal depth (Scenario A: r = −0.542, *p* < 0.01; Scenario B: r = −0.508, *p* < 0.01).

Finally, there was a marginally significant positive correlation between the mean EDA and brake pedal depth (Scenario A: r = 0.334, *p* = 0.071). The correlation analysis results for Scenario A and Scenario B are shown in Table 4.

### 3.2. ANCOVA

#### 3.2.1. Analysis of First Fixation Time on AOI

**Figure 5:**
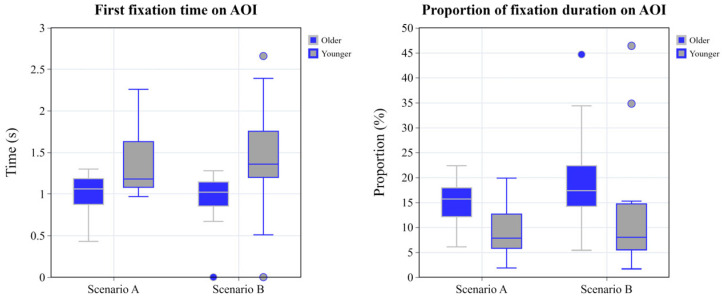
Grouped box plot of first fixation time and proportion of fixation duration on AOI.

The ANCOVA revealed that the main effect of the group was significant, F = 9.619, *p* = 0.04, and η^2^ = 0.263. The results are presented in Table 5 and Figure 5.

#### 3.2.2. Analysis of Proportion of Fixation Duration on AOI

The ANCOVA revealed that the main effect of the group was significant, F = 5.141, *p* = 0.032, and η^2^ = 0.160. The results of the two-factor ANOVA are presented in Table 6 and Figure 5.

#### 3.2.3. Analysis of Braking Reaction Time

**Figure 6:**
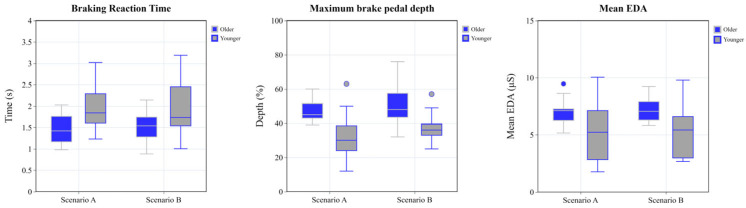
Grouped box plot of braking reaction time, maximum pedal depth, and mean EDA.

The ANCOVA revealed that the main effect of the group was significant, F = 10.476, *p* = 0.003, and η^2^ = 0.280. The results are presented in Table 7 and Figure 6.

#### 3.2.4. Analysis of Maximum Depth of Brake Pedal

The ANCOVA revealed that the main effect of the group was significant, F = 17.659, *p* < 0.01, and η^2^ = 0.395, with the results shown in Table 8 and Figure 6.

#### 3.2.5. Analysis of Physiological Data Analysis

Gaussian filtering was applied to the EDA data to minimize the effects of noise and interference, and then, the mean EDA was calculated based on the built-in statistical tools of Ergolab 3.0. Following this preprocessing, a significance analysis was conducted on the filtered data. The main effect of the group was significant, F = 5.957, *p* = 0.021, and η^2^ = 0.181. The results are presented in Table 9 and Figure 6.

### 3.3. Regression Analysis

Linear regression models were employed to analyze data from Scenario A and Scenario B (Scenario A: β = 0.783, *p* < 0.001; Scenario B: β = 0.675, *p* < 0.001), accounting for 56–58% variance (R^2^ = 0.560–0.581). The mean EDA showed no significant predictive power (*p* > 0.05). The results are presented in Table 10.

## 4. Discussion

Correlation analyses revealed three main findings. First, the brake pedal reaction time was significantly and negatively correlated with the maximum brake pedal travel depth (Scenario A: r = −0.542, *p* < 0.01; Scenario B: r = −0.508, *p* < 0.01), demonstrating that shorter reaction times correspond to a greater braking depth. This relationship suggests the drivers’ behavioral pattern of rapid reaction and forceful braking in emergencies. Second, earlier visual fixation on the critical hazard area (AOI) facilitated faster initiation of braking responses. Third, EDA exhibited a positive trend toward a correlation with the brake pedal depth (Scenario A: r = 0.334, *p* = 0.071), suggesting that higher physiological arousal may be associated with deeper braking and a facilitatory effect on driving performance.

ANCOVA was conducted on all metrics. The results showed that driving experience, as a covariate, had no significant effect on any of the metrics, and the interaction effects between groups and scenarios were also non-significant. However, significant main effects of age group emerged. The older group demonstrated significantly earlier first fixations on the AOI than the younger group (F (1, 27) = 9.619, *p* < 0.05, η^2^ = 0.263). Older drivers allocated a higher proportion of the fixation duration to the AOI (F (1, 27) = 5.141, *p* < 0.05, η^2^ = 0.160), reflecting greater attention to potential hazards across both emergency scenarios. Older drivers exhibited significantly shorter braking reaction times (F (1, 27) = 10.476, *p* < 0.01, η^2^ = 0.280), greater maximum brake pedal depths (F (1, 27) = 17.659, *p* < 0.001, η^2^ = 0.395), and higher mean EDA (F (1, 27) = 5.957, *p* < 0.05, η^2^ = 0.181), indicating superior hazard detection and more rapid evasive planning.

Regression analyses confirmed that, across both scenarios, the latency of the first fixation on the AOI significantly predicted the braking reaction time: earlier fixation was associated with faster braking, underscoring the critical role of attentional allocation in driving safety. Collectively, older drivers adopted safer strategies when confronted with intruding e-bikes or pedestrians.

Unexpectedly, the older group demonstrated safer driving behaviors during the experiment, which differs from many previous studies. First, this divergence primarily results from the simplicity of the emergency scenarios. The experiment employed a straight road section with minimal distractions and isolated, well-defined hazard events. This significantly reduced the cognitive load compared to the complex environments (e.g., intersections, dense traffic, multi-tasking demands) often used in studies reporting age-related challenges. This low-demand setting potentially mitigated the impact of age-related declines in information processing speed or divided attention. It allowed older drivers to effectively focus their preserved cognitive resources on detecting and responding to the single, unambiguous hazard source, enabling their cautious strategies to excel. This reversal underscores the critical role of environmental complexity in modulating age effects. Despite the abrupt emergence of hazards, a buffer distance of approximately 50 m was provisioned for driver response. This design resulted in lower perceived urgency among drivers, thereby helping mitigate adverse effects on older participants.

Importantly, all recruited older drivers were aged 60–70 years (mean = 65 years), exhibiting good health, normal vision, and rich life experience. As young-old drivers at the initial stage of age-related decline, they demonstrate only marginal deterioration in hazard perception, visual processing, and vehicle control. Their heightened caution may thereby enhance driving performance-a pattern aligning with Qiu et al. [19].

Similarly, these findings align with Hakamies-Blomqvist et al. [26], who demonstrated that older drivers can achieve superior safety through prudent driving strategies in specific contexts. This phenomenon primarily stems from three interconnected mechanisms: First, gradual cognitive decline allows extended adaptation periods, enabling older drivers to develop compensatory behaviors-consistent with Charlton et al.’s observation that when driving tasks are more challenging, older drivers engage less in non-driving-related activities (such as scratching their hair, talking/singing, and manipulating the vehicle control panel) to self-regulate [27,28]. Second, through the strategic allocation of visual attention to roadside zones and elevated environmental scanning frequency, they achieved accelerated detection of potential hazards [29,30]. Third, older drivers displayed heightened sensitivity to safety threats, dedicating prolonged focus to risk assessment during emergencies. These findings contribute to mitigating societal concerns regarding young-old drivers’ capabilities. While bolstering driver confidence and alleviating anxiety, our results further suggest that technologies supporting compensatory strategies-particularly assistive systems designed for older drivers-could effectively mitigate the age-related cognitive load during driving.

However, this study has limitations requiring further research. On the one hand, a post hoc power analysis was performed to evaluate the statistical power of the study. The analysis revealed a statistical power of 0.353 under the conditions of an effect size of 0.3, a total sample size of 30, and a significance level (α) of 0.05, which still presents exploratory significance, although the effect of this indicator is not high. Future studies should employ larger sample sizes to obtain more robust driving characteristic data. On the other hand, future studies could incorporate additional metrics to provide a more detailed and multidimensional description and analysis of the differences in driving behaviors between older and younger drivers.

## 5. Conclusions

The findings indicate that older drivers, after recognizing the potential risks associated with their physical decline, were able to drive more safely than younger drivers in simple driving tasks. Specifically, the study identified three key aspects of their performance: First, older drivers maintained higher situational awareness during driving and were able to focus on potential hazards earlier. Second, once a hazard was detected, they were able to initiate braking actions more quickly. Third, they applied greater braking force compared to younger drivers, enabling the vehicle to decelerate more effectively. However, it is important to note that due to the particular experimental methods and scenario settings employed in this study, as well as the limitations imposed by the sample size, caution should be exercised when extrapolating these findings to real-world traffic environments.

## Figures and Tables

**Table 1:** Eye movement metrics and their meaning.

Research Metrics	Meaning
First fixation time on the AOI	The time elapsed from the moment a hazard appears in the driver’s field of view to the moment the driver first fixates on the hazard. This metric reflects the differences in visual attention among different research subjects.
Proportion of fixation duration on the AOI	The ratio of the duration during which the fixation points fall within the AOI to the total duration of the study segment. This metric reflects the degree of interest the participant has in the information within the AOI.

**Table 2:** Driving behavior metrics and their meaning.

Research Metrics	Meaning
Braking reaction time	This indicator reflects the elapsed time from the onset of an emergency scenario to the moment the driver initiates braking action
Maximum brake pedal depth	This indicator can reflect the driver’s sensitivity to driving risks

**Table 3:** Physiological metric and its meaning.

Research Metric	Meaning
Mean EDA	Higher levels of mean EDA are associated with heightened sensitivity, tension, and focused attention. Conversely, lower levels of mean EDA are indicative of a more relaxed state and a balanced mindset.

**Table 4:** Correlation analysis results.

Variable Pair	Scenario A	Scenario B
Brake reaction time–first fixation time	0.739 **	0.741 **
Brake reaction time–maximum brake depth	0.508 **	0.542 **
Maximum brake depth–mean EDA	0.036	0.334
Proportion of fixation duration on AOI–mean EDA	—	0.367 *

Note: * *p* < 0.05, ** *p* < 0.01; *N* = 30.

**Table 5:** Tests of between-subjects effects for first fixation time on AOI.

Source	Type III Sum of Squares	df	Mean Square	F	Sig.	Partial η^2^
Years of driving exp.	0.322	1	0.322	1.034	0.318	0.037
Group	2.993	1	2.993	9.619	0.004	0.263
Groups × Scenarios	0.091	27	0.091	1.128	0.298	0.04
Error	8.401	27	0.311			

**Table 6:** Tests of between-subjects effects for proportion of fixation duration on AOI.

Source	Type III Sum of Squares	df	Mean Square	F	Sig.	Partial η^2^
Years of driving exp.	55.146	1	55.146	0.762	0.390	0.027
Group	371.972	1	371.972	5.141	0.032	0.160
Groups × Scenarios	2.189	27	2.189	0.025	0.875	0.001
Error	1953.675	27	72.358			

**Table 7:** Tests of between-subjects effects for braking reaction time.

Source	Type III Sum of Squares	df	Mean Square	F	Sig.	Partial η^2^
Years of driving exp.	0.129	1	0.129	0.348	0.560	0.013
Group	3.873	1	3.873	10.476	0.003	0.280
Groups × Scenarios	0.020	27	0.020	0.131	0.720	0.005
Error	9.981	27	0.370			

**Table 8:** Tests of between-subjects effects for maximum depth of brake pedal.

Source	Type III Sum of Squares	df	Mean Square	F	Sig.	Partial η^2^
Years of driving exp.	58.154	1	58.154	0.427	0.519	0.016
Group	2402.943	1	2402.943	17.659	<0.01	0.395
Groups × Scenarios	68.625	1	68.625	1.248	0.274	0.044
Error	3673.979	27	136.073			

**Table 9:** Tests of between-subjects effects for mean EDA.

Source	Type III Sum of Squares	df	Mean Square	F	Sig.	Partial η^2^
Years of driving exp.	2.476	1	2.476	0.354	0.557	0.013
Group	41.665	1	41.665	5.957	0.021	0.181
Groups × Scenarios	0.046	1	0.046	0.220	0.643	0.008
Error	188.838	27	6.994			

**Table 10:** Linear regression results.

Scenario	Predictor	β	*p*	R^2^
A	First Fixation Time	0.783	<0.001	0.560
	Mean EDA	0.124	0.371	
B	First Fixation Time	0.675	<0.001	0.581
	Mean EDA	−0.190	0.166	

## Data Availability

The datasets generated and/or analyzed during the current study are not publicly available as data use requires permission from experimental collaborators but are available from the corresponding author on reasonable request.
